# Crucial Role of Lamin A/C in the Migration and Differentiation of MSCs in Bone

**DOI:** 10.3390/cells9061330

**Published:** 2020-05-26

**Authors:** Natividad Alcorta-Sevillano, Iratxe Macías, Clara I. Rodríguez, Arantza Infante

**Affiliations:** Stem Cells and Cell Therapy Laboratory, Biocruces Bizkaia Health Research Institute, Cruces University Hospital, Plaza de Cruces S/N, Barakaldo, 48903 Bizkaia, Spain; Natividad.alcortasevillano@osakidetza.eus (N.A.-S.); iratxe.maciasgarcia@osakidetza.eus (I.M.)

**Keywords:** lamin A/C, mesenchymal stem cells (MSCs), migration, differentiation, mechanosensing, bone formation, bone disease

## Abstract

Lamin A/C, intermediate filament proteins from the nuclear lamina encoded by the *LMNA* gene, play a central role in mediating the mechanosignaling of cytoskeletal forces into nucleus. In fact, this mechanotransduction process is essential to ensure the proper functioning of other tasks also mediated by lamin A/C: the structural support of the nucleus and the regulation of gene expression. In this way, lamin A/C is fundamental for the migration and differentiation of mesenchymal stem cells (MSCs), the progenitors of osteoblasts, thus affecting bone homeostasis. Bone formation is a complex process regulated by chemical and mechanical cues, coming from the surrounding extracellular matrix. MSCs respond to signals modulating the expression levels of lamin A/C, and therefore, adapting their nuclear shape and stiffness. To promote cell migration, MSCs need soft nuclei with low lamin A content. Conversely, during osteogenic differentiation, lamin A/C levels are known to be increased. Several *LMNA* mutations present a negative impact in the migration and osteogenesis of MSCs, affecting bone tissue homeostasis and leading to pathological conditions. This review aims to describe these concepts by discussing the latest state-of-the-art in this exciting area, focusing on the relationship between lamin A/C in MSCs’ function and bone tissue from both, health and pathological points of view.

## 1. Introduction

The nuclear lamina, a thin protein network lining the inner surface of the nuclear envelope, is primarily composed of type V intermediate filaments known as A- and B-type lamins. In mammals, the two major isoforms of A-type lamins are generated by the alternative splicing of the *LMNA* gene: lamin A and lamin C, which are mainly expressed in differentiated cells. B-type lamins, lamin B1 and lamin B2, encoded by *LMNB1* and *LMNB2* respectively, are constitutively expressed in most cell types [1]. Soon after being synthesized, lamin A and B-type lamins undergo sequential post-translational modifications based on their C-terminal CaaX motif (C: Cys, a: an aliphatic residue, X: usually a Met) which functions as a substrate where farnesylation and carboxy-methylation take place. After this complex process, mature B-type lamins retain a farnesyl group at the C-terminal extreme, whereas mature lamin A loses it along with 15 amino acids of the C terminus [2]. This farnesyl group has a role in targeting newly synthesized cytoplasmic lamins to the nuclear envelope, by enhancing the hydrophobic interactions of lamins with the inner nuclear membrane [3]. However, this farnesylation is not always indispensable for the nuclear recruitment of lamins: lamin C is localized to the inner nuclear envelope although it does not contain the CaaX motif to be farnesylated [4]. Regarding the structural organization of lamins within mammalian nuclei, super-resolution microscopy techniques showed that lamin A and lamin B form independent but interacting filament networks adjacent to the inner nuclear membrane [5,6,7,8]. More recently, this observation has been tuned by two studies: not only has the existence of independent lamin A and lamin B networks been corroborated (showing only 18% of co-localization between the A- and B-type lamins), but also a distinct spatial organization of lamins. Thus, in mouse embryonic fibroblasts (MEFs) and human cells (HeLa, fibroblasts), lamin A and lamin B1 form concentric but overlapping networks. In this way, lamin B1, taking advantage of its farnesylated C-terminal group, shows a more peripheral localization, closest to the inner nuclear envelope [9,10].

The nuclear lamina has been shown to undertake two main cellular functions: (1) an essential structural role, providing the shape, and mechanical properties to the nucleus, and (2) as a regulator of gene expression, by modulating chromatin organization and the accessibility of signaling molecules and transcription factors to target promoters [1,11,12]. Recently, nuclear lamina has been shown to be an essential mediator of mechanosignaling, that is, the transduction of exterior physical forces into the nucleus to generate a biological response, which is essential to help the cells adapting to the continuously changing microenvironment [13]. Thus, nuclear lamina components have been shown to be the linkers between the mechanosignals transduced from the cytoskeleton to the nucleus, with lamin A/C executing an essential role in this process [14,15,16]. Indeed, this mechanosensing regulated by lamin A/C has been proposed to be the bridge integrating both the aforementioned structural and gene expression function mediated by lamin A/C [17]. Interestingly, the stoichiometry of the lamin A:B differs depending on the cell types, in fact the relative abundance of lamin A has been shown to scale with tissue and nuclei stiffness [18]. Thus, cells with a high content of A-type lamins exhibit high viscous and stiff nuclei [19], which hamper their migration capacity. On the other hand, cells expressing very low levels of lamin A and C, such as embryonic stem cells, display easily deformable nuclei [20]. Interestingly, bone tissue, which is of mesenchymal origin, has the highest rate of collagen content and thus the highest A:B ratio [18]. Mechanical signals and extracellular matrix (ECM) composition play an important role in bone homeostasis. Indeed, bones are known to respond to mechanical loading, such as exercise, to promote osteo-anabolic pathways [21]. Mesenchymal stem cells (MSCs) are the natural progenitors of osteoblasts, the bone forming cells. MSCs undergo the multi-step process of osteogenesis in response to different cues (of both biochemical and mechanical nature) coming mainly from the surrounding ECM [22,23]. Moreover, in the bone healing process, inflammatory mediators activate and mobilize tissue-resident, endogenous MSCs which migrate from their niche to the damaged site in order to facilitate bone tissue regeneration [24]. To achieve both migration and osteogenic differentiation, MSCs must reorganize their nuclear lamina shape and/or composition, with lamin A orchestrating this process. Thus, levels of lamin A are known to be increased during osteogenesis while they are decreased during adipogenesis of MSCs [18,25]. In the same way, there are pathological conditions affecting lamin A/C, such as physiological aging and *LMNA* mutations, which will have a negative impact in migration and osteogenesis of MSCs, affecting bone tissue homeostasis. 

In this article, we will review the current knowledge about the crucial role of lamin A/C in regulating biological processes in MSCs which are critical to ensure a proper bone tissue homeostasis.

## 2. Lamin A/C and Bone Formation

Bone is a tissue in a constant remodeling, which is capable of self-repairing after an injury. Bone fracture healing can occur through two different mechanisms: intramembranous and endochondral bone formation. During intramembranous ossification, MSCs proliferate and condense into compact nodules called nidus. Here, MSCs differentiate into osteoprogenitor cells to finally become osteoblasts. Then, osteoblasts start secreting an extracellular matrix rich in type I collagen, which is able to bind calcium salts. Eventually, the extracellular matrix becomes calcified, forming the osteoid. Normally, osteoblasts are separated from the osteoid but some cells can be trapped into the calcified area, becoming osteocytes. The process continues with the growth of bone spicules from the osteoid. Lastly, the entire region is surrounded by compact mesenchymal cells to form the periosteum membrane. This ossification process occurs in flat bones of the face, most of the skull bones and clavicles.

On the other hand, endochondral bone formation requires the presence of cartilage and occurs in the tissue adjacent to the fracture and surrounding soft tissues. Through this mechanism, the long bones of the axial (spine and ribs) and appendicular (limbs) skeleton are formed. The beginning is similar to the previous process, with MSCs grouping together where the new bone will take shape. However unlike the former, cells differentiate into chondrocytes that will proliferate, synthesizing their proper extracellular matrix rich in type II collagen and proteoglycans. The cartilaginous tissue forms a soft callus that stabilizes the fracture area, serving as a mold for the newly forming bone. Subsequently, chondrocytes stop dividing and become hypertrophic chondrocytes. They modify the ECM secreting type X collagen and fibronectin, promoting the mineralization of the tissue by calcium deposition. At that point, the blood vessels invade the tissue in order to vascularize it, meanwhile hypertrophic chondrocytes begin to suffer apoptosis. Certain hypertrophic chondrocytes survive and can differentiate into osteoblasts [26]. The MSCs surrounding the area differentiate into osteoblasts, furthermore beginning to synthesize extracellular bone matrix rich in type I collagen. This process will proceed until all cartilaginous tissue is replaced by bone tissue.

Usually, both types of ossification are combined during fracture healing and bone repair, after bone injury [27]. In order to achieve an accurate bone remodeling, everything must be closely regulated by different factors, such as growth, differentiation and transcription factors [28,29]. Therefore, bone formation can be measured in vitro and in vivo by different markers, from transcription factors such as osteocalcin (OCN), osterix (OSX) and bone sialo-protein (BSP), to akaline phosphatase expression and calcium deposition by Alizarin Red staining. The influence of lamin A/C in bone formation has been assessed in vitro in several studies. In lamin A/C-inhibited MSCs and mature osteoblasts, a significant reduction in alkaline phosphatase and Alizarin red staining have been shown, confirming a significant reduction in osteoblast differentiation and mineralization. Additionally, significantly lower levels of OCN, OSX and BSP were measured in *LMNA* knocked-down cells [30]. Moreover, during bone remodeling, MSCs’ differentiation into osteoblasts (osteoblastogenesis) and induction and maturation of osteoclasts (osteoclastogenesis) were altered by the absence of lamin A/C in MSCs: while osteoblastogenesis was inhibited (measured by a reduction of osteocalcin secretion and alkaline phosphatase expression), osteoclastogenesis was enhanced [31]. In fact, lamin A/C modulates the expression of the two osteoclastogenesis-regulating proteins, receptor activator of nuclear factor κ-B ligand (RANKL) and osteoprotegerin (OPG): RANKL is expressed by immature osteoblasts, thus osteoblast differentiation arrest due to lamin A/C inhibition leads to increased expression of RANKL levels, unbalancing the RANKL/OPG ratio, inducing an osteoclastogenic environment. Conversely, lamin A overexpression in mouse pre-osteoblastic MC3T3-E1 cells has been shown to promote osteoblast differentiation and calcification by inducing the expression of: alkaline phosphatase, type I collagen, BSP, OCN and dentin matrix acidic phosphoprotein 1 (DMP-1), in the presence of bone morphogenetic protein 2 (BMP-2) growth factor [32]. Taken together, all this evidence reveals the crucial role lamin A/C plays in bone remodeling.

## 3. Lamin A/C and MSCs

Adult MSCs are spindle-shaped cells which use has been expanded to cell therapy, regenerative medicine and tissue repair, due to the beneficial properties that they present: self-renewable, multipotent, immunomodulation [33], easily accessible and culturally expandable in vitro, genomic stability and few ethical issues [34]. Moreover they have been isolated from many different sources [35], such as bone marrow and adipose tissue in addition to perinatal sources such as umbilical cord blood [36]. The International Society for Cellular Therapy has proposed minimum criteria to define MSCs [37]: They (i) should exhibits plastic adherence (ii) possess specific cell surface markers such as cluster of differentiation (CD)73, CD90, CD105 and lack expression of CD14, CD34, CD45 and human leucocyte antigen-DR (HLA-DR), besides, (iii) they should present the ability to differentiate in vitro into adipogenic, chondrogenic and osteogenic lineages. 

These cells are gaining interest due to the benefits that are presenting when used in therapy, urging to delve into their potential and mechanisms of action [38]. They have been proposed to treat several diseases, showing promising results for repairing damaged tissues, both in animal models and in human clinical trials [39,40,41]. MSCs have demonstrated homing ability: the capacity to migrate into specific tissues and differentiate into chondrogenic, osteogenic or adipogenic lineage. Moreover, they secrete chemokines, cytokines and growth factors crucial in tissue regeneration [42]. The processes of MSCs’ migration to injured tissue and differentiation into specific lineage are paramount in bone formation and remodeling. These processes are highly determinate by lamin A/C implication for their correct accomplishment [43].

### 3.1. MSCs’ Migration

The process by which MSCs migrate to their target tissue has been deeply studied, since it is a vital requirement to later on differentiate and form new bone. Several studies have demonstrated that the migration of these cells is mainly affected by two types of factors: chemical and mechanical ones. 

#### 3.1.1. Chemical Factors Involved in MSCs’ Migration

The chemical factors are part of the microenvironment in which MSCs reside, playing a key role in the migration of these cells to injured tissue sites. The first step in bone formation requires MSCs to migrate and differentiate in the place where the injury was produced. MSCs migrate toward various signals, including growth factors, chemokines and cytokines [44], factors that are released by several immune cells (neutrophils, macrophages, lymphocytes and others) during the initial acute inflammatory phase of fracture healing [45]. Tan et al. [46] uncovered that platelet-derived growth factors (PDGFs) promoted endogenous MSCs’ migration to the fracture from remote sites. Specifically, PDGF-AA regulates MSCs’ migration and osteogenic differentiation via the BMP-Smad1/5/8 signaling pathway [47]. Secretion of tumor necrosis factor-α (TNF-α) by macrophages, immune inflammatory cells, and even by the MSCs present in the periosteum [48], has shown to induce osteogenic differentiation of MSCs in vitro [49]. Also, TNF-α-activated MSCs suppress inflammation by inducing Interleukin (IL)-10 production in macrophages [50], demonstrating the regulatory role of MSCs in the bone healing process. The downregulation of the pro-inflammatory response is fundamental for bone repair, since systemic and maintained inflammation is detrimental for fracture healing accomplishment [51].

During bone remodeling, transforming growth factor-β1 (TGF-β1) has been shown to play a role in the recruitment of MSCs to the bone resorption area. TGF-β1 is a cytokine present in bone extracellular matrix, normally in its inactive form through non-covalent binding to latency-associated protein. Thus, the receptor-binding domains of the active TGF-β1 are hidden by this union. This cytokine, crucial during the bone resorption process, is activated and released, favoring the migration of osteogenic MSCs through the SMAD signaling pathway to resorptive sites [52].

Another factor involved in bone remodeling is Sry-related high-mobility group box 9 (SOX9). This transcription factor regulates sex determination, chondrocyte differentiation and other developmental events. In fetal and juvenile growth plates, SOX9 is expressed in resting and proliferating chondrocytes, being maximal in pre-hypertrophic chondrocytes. However, in the hypertrophic zone, it disappears completely [53]. Hattori et al. demonstrated that SOX9 downregulation in the hypertrophic zone of the growth plate is a necessary event to allow cartilage vascularization, cartilage resorption and formation of trabecular bone [54].

#### 3.1.2. Mechanical Factors Involved in MSCs’ Migration

From a mechanical point of view, cells have to be able to deform in order to migrate into specific tissue to then differentiate and form new bone—a process highly regulated by the cytoskeleton [55]. Nevertheless, the nucleus is the largest, stiffest organelle in the cell; hence, it is the primary impediment to motion as cells squeeze through tight spaces. When the nucleus is too stiff to be pushed or pulled through a small opening, the migration is limited. Conversely, when nuclear softness and elasticity increases, cell migration is promoted. Therefore, a deformable nucleus facilitates MSCs’ migration as it accommodates motion of the cell through three-dimensional (3D) environments.

The nuclear shape and stiffness have been shown to be regulated by both lamin B1 and lamin A/C [9,56,57]. In the case of lamin A/C, several studies have demonstrated that cells which ectopically express lamin A/C have stiffer nuclei that resist deformation [56,58,59], limiting migration. Conversely, depletion of lamin A/C results in irregularly shaped nuclei and severely reduces nuclear stiffness [15,58,60,61,62]. Indeed, low lamin A levels increase MSCs’ migration, as it was demonstrated via lamin A knockdown by ∼50% [19]. However, not only the lamin A/C content, but also the lamin A/C to lamin B1 ratio will influence the migration capacity of cells. Indeed, MSCs have shown slower migration through two-dimensional (2D) and 3D microenvironments than other primary human cells derived from mesodermal origin, due to a high lamin A/C to lamin B1 ratio that increases nuclear rigidity [63].

**Figure 1:**
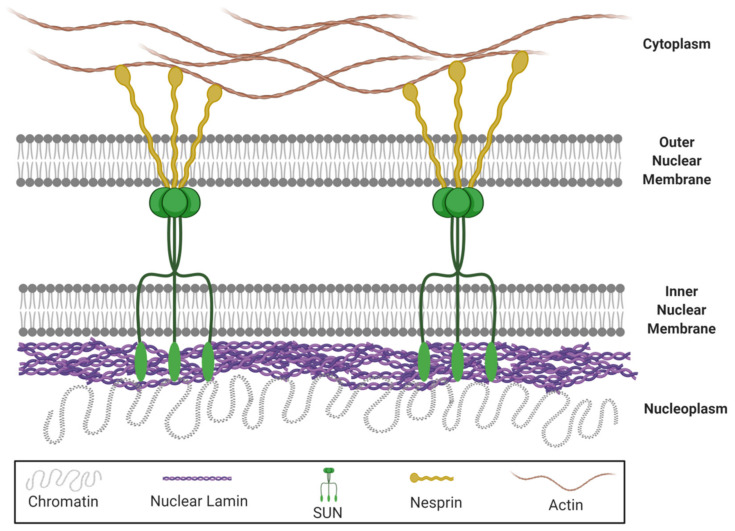
Linker of Nucleus and Cytoskeleton (LINC) complex. This structure consists of SUN (Sad1 and UNC-84) proteins anchored in the inner nuclear membrane and nesprins anchored in the outer nuclear membrane. While nesprins interact with different proteins of the cytoskeleton such as actin, SUN proteins are linked with lamins that surround the nuclear envelope.

Besides, lamin A/C is implicated in the nuclear-cytoskeletal coupling, which plays a vital role in the nuclear mechanotransduction and determines the mechanical properties of nuclei [64,65]. In fact, mutations in lamin A/C disturb nuclear-cytoskeletal coupling in *LMNA*-/- mice [66]. The nucleus is mechanically connected to the rest of the cell, via Linker of Nucleus and Cytoskeleton (LINC) complex structures which are located in the nuclear envelope [67,68]. This complex consists of SUN (Sad1 and UNC-84) proteins anchored in the inner nuclear membrane and nuclear envelope spectrin-repeat-containing proteins (nesprins) anchored in the outer nuclear membrane (Figure 1). In that way, the LINC complex connects the cytoplasmic cytoskeleton with the inner nucleoskeletal lamin A/C network [68,69], allowing the reorganization of lamins in response to mechanical stress [70]. Therefore, it is responsible for limiting nuclear deformation [67].

Then, there are different mechanical factors that could affect the migration of MSCs through the influence of lamin A/C, such as mechanical strain, matrix stiffness [71], 3D geometry and gravity.

Different in vitro and in vivo studies have shown that the migration of MSCs is affected by mechanical strain. Mechanical strain has the ability to increase human MSCs migration, as it was demonstrated when static mechanical tension at 5% (for 6 h) [72] or 10% (for 12 h) [73] was applied. Moreover, cyclic mechanical stretching with 10% amplitude at 1 Hz frequency (for 8 h) promotes bone marrow MSCs’ (BMSCs) migration but reduces BMSCs’ invasion [74]. Cell invasion is a feature typical of malignant metastatic cells that actively invade a surrounding tissue, usually attracted by a chemoattractant gradient. This process, unlike cell migration, involves the secretion of specific proteases to destroy the ECM of the target tissue in order to facilitate its entrance, altering the structure of the receptor tissue. As we have previously reported, lamin A/C is closely implicated in the transmission of mechanical stress through the nucleus. Lamin A/C-deficient fibroblasts are characterized by defective nuclear mechanics and mechanotransduction under mechanical strain. That was reflected by increased nuclear deformations and nuclear fragility, attenuated expression of mechanosensitive genes, as well as impaired transcriptional activation leading to impaired viability of mechanically strained cells [60,75]. Overall, the involvement of lamin A/C in mechanical stress will influence MSCs’ migration. 

Regarding matrix stiffness, MSCs are surrounded by an ECM that transmits complex biochemical [76] and biophysical signals [77]. The physical signals provided to MSCs could regulate their migration, so different studies have focused on the effect of matrix stiffness and elasticity [19,78]. Raab et al. demonstrated that MSCs migrate to stiffer portions of the substrates (from a soft matrix (1 kPa) to a stiff matrix (34 kPa)), by polarizing the cytoskeleton [77]. The assembled microtubule network was necessary for directed migration of MSCs [79]. Since lamin A/C is tightly linked to the cytoskeleton via the LINC complex, lamin A/C is also implicated in how cells respond to matrix stiffness [80]. In fact, lamin A/C levels control nuclear strain stiffening at large deformations, that is, during cell migration [81].

To deepen the understanding of the behavior of MSCs in tissue regeneration, it is mandatory to study how 3D geometry could influence it. It is known that the role of surface curvature is related with the migratory capacities of MSCs. Werner et al. demonstrated that cells present the ability to migrate faster on concave spherical surfaces, compared to flat and convex spherical surfaces, as a consequence of a reduced contact between the cell and the material surface [82]. Moreover, the nuclei of cells on convex surfaces present high lamin A levels, suggesting that these cells withstand high intracellular tensions, so the migration of MSCs is obstructed. These observations indicate that the 3D substrate curvature influences cell attachment morphology, thus modulating the cytoskeletal forces acting on the nucleus. 

Another factor that affects MSCs’ migration is gravity. Little is known about the cellular reaction to simulated microgravity conditions in vitro. Mao et al. reported that stimulated microgravity (rotated at 10 rpm, approximately 1 × 10^−3^ g) inhibited the migration of rat BMSCs via reorganizing F-actin and increasing cell stiffness [83]. A recent report has studied how microgravity affects the migration of MSCs and the implications of lamin A/C. MSCs cultured under normal conditions present a stretched morphology and undergo unidirectional migration; on the contrary, cells cultured under simulated microgravity conditions undergo multidirectional migration, with higher frequency of directional changes of cell movement. Observing the cytoskeleton, cells grown under conventional culture conditions had longitudinal actin stress fibers along both the apical and basal sides. Alternatively, the cells grown under simulated microgravity conditions presented larger cortical actin fibers in the filopodia and lamellipodia regions and had less stress fibers located mainly along the basal side. Nevertheless, lamin A/C was mainly located on the apical side in cells under conventional culture conditions, indicating basal-to-apical polarization. Conversely, cells cultured under simulated microgravity conditions showed lamin A/C localization on both the apical and basal sides. Taken together, gravitational changes might induce alternations in the nuclear lamina organization and nuclear lamina–cytoskeleton interactions, inducing mechanotransduction changes in human MSCs [84]. 

All in all, MSCs’ migration to the target tissue is regulated by chemical factors such as growth factors, chemokines and cytokines. In addition, mechanical factors play a key role in MSCs’ migration. First of all, nucleus stiffness, which is determined by lamin A/C levels, is the main regulator during migration. Hence, the lamin A/C to lamin B1 ratio increases nuclear rigidity, thereby slowing down the migration of MSCs. Moreover, lamin A is implicated in the nuclear-cytoskeletal coupling via the LINC complex, allowing the reorganization of lamins in response to mechanical stress. There are different mechanical factors that could affect the migration of MSCs through the influence of lamin A/C. In summary, increased migration of MSCs is obtained with mechanical strain, concave spherical surfaces and under microgravity conditions.

### 3.2. MSCs’ Differentiation

Once MSCs have migrated to their target tissue, they differentiate into osteogenic, chondrogenic or adipogenic lineages. At this point, microenvironment is essential to determinate cell fate, and both biochemical and mechanical cues are involved in this process. Moreover, the nuclear envelope protein lamin A plays a key role in MSCs’ differentiation, since it interacts with different pathways involved. Different mutations in the *LMNA* gene alter the expression profile of MSCs during differentiation, in a mutation-specific manner. Thus, each *LMNA* mutation promotes a unique expression pattern of genes involved in a lineage-specific differentiation and this pattern is shared by the phenotype-specific mutations [85]. 

#### 3.2.1. Response of MSCs to Biochemical Cues

Cellular and molecular signaling pathways, in addition to microenvironmental changes, have been studied in order to understand the role of cytokines, growth factors, extracellular matrix molecules and transcription factors [86] that regulate the differentiation of MSCs toward a specific cell type. Lamin A/C has the capacity to directly regulate the response of MSCs to biochemical cues, by interacting with those factors involved. 

The Wnt/β-catenin signaling pathway plays a central regulatory role in differentiation of MSCs. In the canonical Wnt signaling pathway, Wnt binds to different receptors (Frizzled family receptors and low density lipoprotein (LDL)-receptor-related protein 5,6) to induce β-catenin stabilization. Subsequently, the stabilized β-catenin translocates to the nucleus and forms a complex with the DNA-binding transcription factors T cell factor/lymphoid enhancer factor to activate a Wnt-controlled gene expression program [87]. These genes are tightly related with MSCs’ differentiation, since the Wnt signaling pathway promotes osteogenic differentiation of MSCs [88]. β-catenin induces differentiation of osteoblasts and osteoblastic matrix production [89] and can also suppress the differentiation of MSCs into adipogenic and chondrogenic lineages [36,90]. Therefore, the Wnt/β-catenin pathway has an essential role in bone regeneration and repair [91]. Regarding the influence of lamin A/C, it physically interacts with β-catenin and facilitates its translocation into the nucleus and its transcriptional activity, thus inducing osteogenesis. In this way, Bermeo et al. demonstrated that overexpression of *LMNA* in human BMSCs leads to increased osteogenic and decreased adipogenic differentiation potential, through the regulation of the Wnt/β-catenin pathway [25].

Notch signaling is a highly conserved pathway that regulates cell fate decisions and skeletal development [92]. In the canonical Notch pathway, the single-transmembrane cell surface receptors undergo sequential proteolytic cleavage upon binding of their ligand. Following binding, the Notch intracellular domain is released from the plasma membrane and translocated into the nucleus, where it interacts with transcription factors to activate transcription of target genes [93,94]. Notch stimulates differentiation of MSCs into osteoblasts [95] and activation of Notch promotes osteogenic differentiation in a tissue-specific, dose-dependent manner [96]. Conversely, its inhibition promotes adipogenic differentiation [97]. In this way, lamin A/C interacts with Notch signaling [98], thereby influencing MSCs’ differentiation. The lamin A–Notch interaction can be realized both through the chromatin regulatory mechanism and through direct structural interactions, for example, via emerin-dependent suppression of Notch signaling [99,100]. Many studies have been reported analyzing the effect of different *LMNA* mutations in the Notch pathway, and thereby in differentiation of human MSCs and mesenchymal origin cells. Bogdanova et al. reported that mutations in the *LMNA* gene may affect the function of Notch signaling in human MSCs, suggesting that the interaction of lamin A/C with Notch signaling components may be one of the mechanisms regulating MSCs’ differentiation. *LMNA* mutations have different effects on the efficiency of MSCs’ osteogenic differentiation and on the expression of specific osteogenic markers [98]. Therefore, the Notch pathway may be involved in the modified cell capacity for differentiation caused by *LMNA* mutations. In addition, Perepelina et al. reported that a specific mutation in *LMNA* (R482L, associated with Dunningan-type familial partial lipodystrophy) contributes to the downregulation of Notch activation in MSCs and decreases adipogenic differentiation when Notch is activated [101]. This mutation leads to nuclear blebbing and alterations in nuclear morphology in fibroblasts. Moreover, emerin binding to mutated lamin was impaired [102], suggesting that interaction with Notch might be mediated by emerin. A recent study has explored the influence of specific *LMNA* mutations (R527C and R471C) on the pro-osteogenic response of human cells of mesenchymal origin, and the interaction of *LMNA* with the Notch pathway. The outcomes obtained have shown a broad range in expression level of Notch-related and pro-osteogenic genes between the different cellular types used in the study, suggesting that the effect of a *LMNA* mutation might be influenced by the intrinsic molecular context of a cell lineage [103]. In conclusion, these studies demonstrate that lamin A/C mutations could have a critical effect on osteogenic differentiation depending on Notch activation and cell type. 

**Figure 2:**
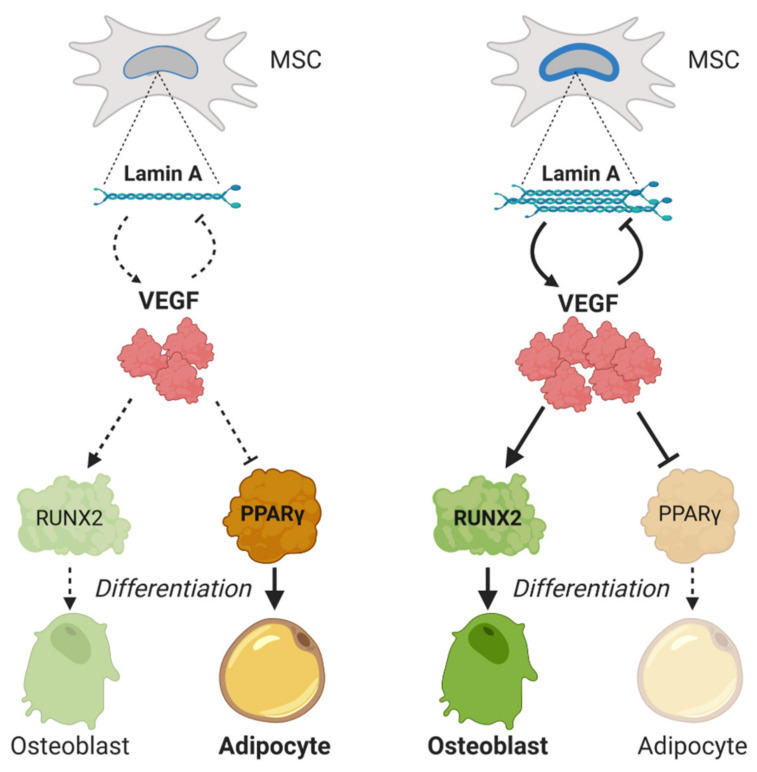
Mesenchymal stem cells’ (MSCs) fate is regulated by lamin A and vascular endothelial growth factor (VEGF) interaction. High levels of lamin A induce the expression of VEGF, which in turn induces the Runt-related transcription factor (RUNX2) transcription factor synthesis, responsible for osteoblast differentiation, inhibiting the adipocyte differentiation. However, low levels of VEGF lead to activation of the peroxisome proliferator-activated receptor (PPAR)-γ transcription factor, responsible of adipogenesis. Dashed lines represent the routes that are downregulated or compromised.

Vascular endothelial growth factor A (VEGFA) is a key regulator in MSCs’ differentiation. Its transcription is downregulated by SOX9, by interaction with regulatory SRY elements in the VEGFA gene [54]. Some studies show a significant decrease of VEGF levels in MSCs with age [104,105]. Accordingly, postmenopausal women with high/low production of VEGF polymorphisms have higher/lower lumbar spine bone mineral density, respectively [106]. Furthermore, the effect of VEGF on osteoblastogenesis seems to be mediated by a functional interaction between VEGF and lamin A/C [107,108]. Heterozygous lamin A-deficient mice express reduced levels of VEGF and Runt-related transcription factor (RUNX2), along with higher levels of peroxisome proliferator-activated receptor (PPAR)-γ (PPARγ). In addition, VEGF knock-down MSCs show the same expression profile, with low RUNX2 and increased peroxisome proliferator-activated receptor (PPAR)-γ. Interestingly lamin A levels are increased when VEGF levels are low, meaning a reciprocal interaction between both molecules (Figure 2).

RUNX2 is also a known downstream transcription factor in Wnt/β-catenin signaling. RUNX2 induces the differentiation of MSCs into immature osteoblasts [109], promoting the expression of osteogenesis-related genes [110], so it is considered an essential transcription factor in osteoblastogenesis. Lamin A/C interacts with RUNX2, the essential transcription factor for osteoblast differentiation [111]. When lamin A/C was knocked down in mature osteoblasts, reduced expression levels of RUNX2 were reported [31]. Moreover, downregulation of lamin A/C in MSCs reduces nuclear binding of RUNX2 to osteogenic promoters [30]. The absence of lamin A/C would allow proteins of the nuclear membrane such as inner nuclear membrane protein (MAN-1) to antagonize osteogenic proteins, affecting RUNX2 mobility and activation. MAN-1 is a protein of the nuclear envelope that is closely regulated by lamin A through a direct physical interaction [112]. Moreover, MAN-1 colocalizes with RUNX2. An in vivo study of *LMNA* null mice has associated absence of lamin A/C with an abnormal interaction between RUNX2 and MAN-1 [113]. In the absence of lamin A/C, expression of MAN-1 increases, thus reducing the availability of RUNX2 and its DNA binding. To sum up, the presence of lamin A/C is necessary for correct osteogenesis and consequent bone formation, guaranteeing the correct function of the RUNX2 pathway. 

The nuclear lamina is known to serve as a resting platform for transcription factors, thus restricting their access to chromatin and therefore limiting their activity as activators or repressors of gene expression [114]. In MSCs, alterations in lamin A protein have been described to disrupt nuclear organization, leading to the sequestration of transcription factors to the nuclear periphery [115,116,117,118]. This pathological entrapment of transcription factors leads to an impaired homeostasis in MSCs, affecting their fate [116,119]. This is the case of PPARs, which are essential regulators of MSCs’ differentiation [120]. PPARγ acts as a positive regulator of adipocyte differentiation [121] and its overexpression enhances and accelerates the adipogenic differentiation of MSCs, both in vitro and in vivo [122]. These receptors are also regulated by lamin A/C. PPARγ ligands are normally trapped at the nuclear periphery by lamin A/C, so the lack of lamin facilitates its release, promoting PPARγ activation and inducing adipogenesis. Thus, lamin A/C acts as an inhibitor of adipocyte differentiation, affecting PPARγ signaling [123].

#### 3.2.2. Response of MSCs to Mechanical Cues

Recent studies have reported that the effects of physical/mechanical cues of the microenvironment play an important role in regulating MSCs’ fate [124,125], suggesting that the mechanical properties [126] and the differentiation ability of MSCs are closely linked [127,128]. In the same way as MSCs’ migration, the nucleus of the cell is the main regulator of cell fate, since it regulates gene expression and its capacity to respond to external stimuli. Nuclear shape and stiffness changes during MSCs’ differentiation, until the type of cell required for each specific tissue is obtained. These two properties of the nucleus are closely regulated by lamin A/C [129]. During cell differentiation, nuclear stiffness increases, along with the *LMNA* expression [130]. Conversely, knockdown of the *LMNA* gene in differentiated cells decreases nuclear stiffness [65]. 

The nucleus and the nuclear envelope protein lamin A/C also play a crucial role in cellular mechanotransduction, which are needed to transmit mechanical signals and respond to mechanical forces [131] during differentiation. As we have mentioned before, LINC complex structures mechanically connect the cytoplasmic cytoskeleton with the inner lamin A/C network. This ability is important to respond to external mechanical cues [132], such as mechanical strain, matrix stiffness [133] and 3D geometry. In addition, chromatin is arranged on the internal nuclear scaffold that lamin makes up. So, external mechanical stimuli could regulate heterochromatization [134] via LINC and lamin A mechanotransduction [135], thus regulating the expression of genes involved in MSCs’ differentiation [136]. Moreover, during development, lamin A levels increase to mechano-protect the genome [137]. 

MSCs’ migration is in part regulated by mechanical cues, such as mechanical strain, matrix stiffness and 3D geometry, and the same happens with MSCs’ differentiation. To demonstrate the influence of mechanical strain in MSCs’ fate, MSCs were cultured in highly adipogenic medium and mechanical strain was applied for 6 h daily. Under mechanical strain, expression of adipogenic markers was inhibited and β-catenin nuclear translocation was enhanced, suggesting an induction of osteoblast lineage differentiation [138,139]. Moreover, the effects of mechanical strain on osteogenic and adipogenic differentiation of cultured MSCs were also studied. In MSCs subjected to strain stimulation, levels of osteogenic markers (RUNX2 and OSX) gradually increased, while levels of adipogenic markers (PPAR*γ*-2) and the emergence of lipid droplets decreased. Thus, mechanical strain promotes differentiation of MSCs into osteoblasts and impedes differentiation into adipocytes, clarifying the mechanisms underlying the effects of exercise on bone repair and reconstruction [140]. Since lamin A/C is implicated in mechanotransduction, mechanical strain that MSCs support would influence their differentiation through the envelope protein lamin A/C. 

Matrix stiffness could determine MSCs’ fate, since nuclear shape changes depending on the substrate that is surrounding the cell. On soft substrate, the nuclear envelope is wrinkled and relaxed, whereas, on stiff substrate, the nucleus is flattened by stress fibers and appears tense and smooth [141]. So, remodeling of the nucleus (based on the substrate) could have significant regulatory effects on cell differentiation and function [135]. In this way, lamin A/C levels will be tightly related with matrix stiffness: lamin A/C level increases with tissue stiffness and decreases in soft matrix. Finally, lamin A levels and matrix stiffness regulate MSCs’ differentiation [18]. For adipogenic differentiation, a soft matrix and low lamin A/C levels (or *LMNA* knockdown [142]) are needed. Conversely, high lamin A levels and a hard matrix (Elasticity = 25–40 kPa, at least as pre-calcified bone [22]) is needed for osteoblast differentiation [30]. As mentioned before, this occurs because matrix stiffness tenses the nucleus, increasing lamin A/C content, thus suppressing soft tissue phenotypes such as adipose tissue [143]. 

As occurred in MSCs migration, surface curvature also regulates differentiation, since focal adhesions have the ability to sense the mechanical cues and regulate signaling pathway responses [144]. It has been demonstrated that convex spherical surfaces induce osteogenic marker expression, and thus osteogenic differentiation of MSCs, compared to flat and concave spherical structures. This effect was observed both in cells cultured in expansion medium and in osteogenic medium. Furthermore, equally, F-actin and osteocalcin intensity levels increased with increasing curvature, indicating that cells can also sense and respond to the magnitude of curvature. The level of lamin A/C was quantified in different curvatures: on convex substrates, lamin A/C levels were 2.5× higher compared to concave surfaces, and 1.4× higher compared to flat surfaces. High lamin A/C levels suggest that high intracellular tensions are exerted on the nuclei of cells on convex surfaces. These results show that in addition to substrate stiffness, 3D surface curvature is a further relevant parameter that can change the stress fiber forces on the nucleus, and thus nuclear morphology and lamin A expression [82].

#### 3.2.3. Response of MSCs to Other Mechanosensitive Pathways

New mechanisms by which nuclear lamin A/C mediates cell differentiation have been evaluated [145]: the interaction with other mechanosensitive pathways that tightly regulate differentiation of MSCs. The retinoic acid (RA) pathway plays a role in development and regeneration, in addition to participating in MSCs’ lineage specification [146]. Concerning osteogenesis, it has been demonstrated that RA inhibits differentiation and mineralization of pre-osteoblasts by downregulation of the Wnt/β-Catenin signaling pathway [147]. Lamin A is strongly linked with vitamin A/RA, since lamin A transcription, dephosphorylation and subsequent stabilization are regulated by the RA pathway [18]. How antagonists and agonists of RA receptors (RAR) could regulate lamin A levels and MSCs differentiation has been analyzed. While RAR-specific antagonist increases the expression of lamin A in rigid matrix, RAR-agonist represses its expression. Therefore, RAR-antagonist promotes lamin A-dependent osteogenesis on rigid substrates, with pretreated xenografts calcifying in vivo to a similar extent as native bone. In conclusion, lamin A and the RA pathway are involved in MSCs, sensing substrate stiffness and lineage determination [148]. 

Another pathway that modulates cell fate determination of MSCs is the Yes-associated protein (YAP) and transcriptional coactivator with PDZ-binding motif (TAZ). These proteins are key regulators in cells, perceiving their microenvironments [149] and then determining MSCs’ fate. In response to stiff substrates, YAP1 expression increases, is translocated into the nucleus and osteogenesis in MSCs is enhanced [150]. In this way, the YAP/TAZ pathway plays a crucial role in human adipo-osteogenic differentiation induced by ECM stiffness [151]. Increasing YAP expression enhances osteogenic differentiation but suppresses differentiation to adipocytes. On the contrary, low YAP levels promote adipogenic differentiation but inhibit osteogenic differentiation. However, how lamin A/C affects the YAP/TAZ pathway is still not fully understood. Evidence suggests that mechanosensing through YAP depends on forces transmitted through the cytoskeleton and to the nucleus [152]. In this way, lamin A/C might have crosstalk with the YAP/TAZ pathway mediated by actin filaments. Bertrand et al. demonstrated that human myoblasts with *LMNA* mutations have mechanosensing defects through a YAP-dependent pathway [153]. Moreover, lamin A overexpression in MSCs on stiff matrix produces a decrease in both total YAP1 levels and nuclear localization, but *LMNA* knockdown also resulted in decreased YAP1 levels [18]. Therefore, the results obtained until now do not have a consensus, besides being cell-type-dependent. 

Finally, the serum response factor (SRF)/megakaryoblastic leukaemia 1(MKL1) pathway senses mechanical signals via actin polymerization [154]. Regulation of the transcriptional coactivator MKL1 by actin cytoskeleton dynamics decreases adipocyte differentiation by the inhibition of PPARγ [155]. The same result was obtained with the SRF transcription factor [156]. Moreover, SRF-deficient adult mice presented a marked decrease in bone mineral density and bone formation, whereas SFR-deficient osteoblasts exhibited reduced cell differentiation and mineralization in vitro. The main reason is that SRF deficiency decreased the transcriptional activity of RUNX2, pointing out the relevance of SRF in osteoblast differentiation. On the other hand, SRF indirectly regulates lamin A by nuclear actin binding [157]. It has been reported that lamin A/C promotes SRF/Mkl1-dependent transcription: *LMNA*-deficient mutant cells have shown impaired nuclear translocation and downstream signaling of the mechanosensitive transcription factor MKL1 [158]. Moreover, *LMNA* knockdown suppress the transcription of several components of the SRF pathway [159] as well as enhances adipogenesis [18]. 

### 3.3. MSCs’ Migration versus Differentiation

**Figure 3:**
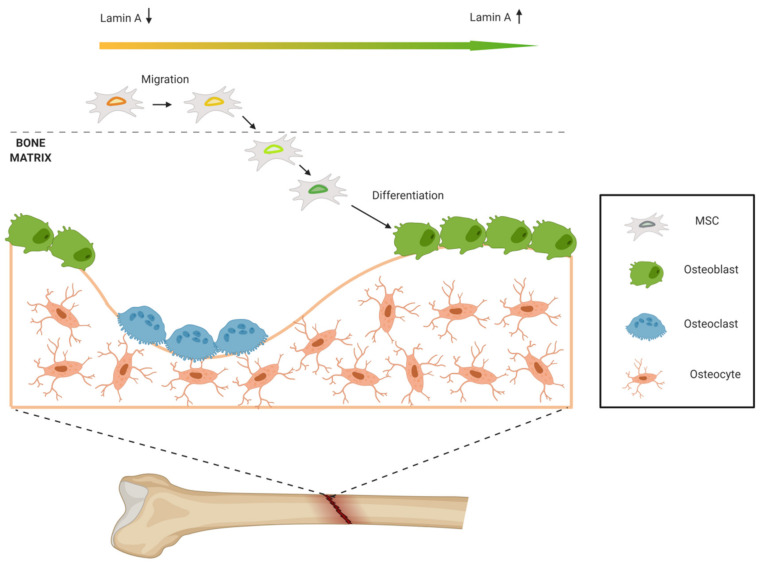
MSCs’ migration and differentiation. Lamin A/C levels in MSCs vary according to the microenvironment. Low lamin A/C levels are needed for migration, while high levels are required for osteogenic differentiation.

It is known that cells with higher migration ability tend to have enhanced differentiation potential, but the optimum amount of lamin A/C is different for MSCs’ migration and differentiation [43]. During bone formation, MSCs migrate until they reach the bone. For MSCs’ migration, soft nucleus allows cell deformation and motion through tight spaces, so low lamin A/C levels are required. Conversely, to promote osteogenic differentiation, high lamin A/C levels are needed. This contrary phenomenon may be the result of mechanical microenvironment modulation. When MSCs reach the bone, they adhere to bone tissue and mechanical signaling changes: the 3D geometry is different, and the rigidity of the matrix is higher. In this way, lamin A/C levels increase, promoting osteogenic differentiation and forming new bone. So, MSCs with high migration ability may be more sensitive to force and able to upregulate lamin A/C in mechanical microenvironment for later osteogenic differentiation (Figure 3).

## 4. Lamin A/C Dysfunction in MSCs, Aging and Bone Disease

### 4.1. Lamin A/C Levels Alterations

As mentioned above, levels of lamin A/C expression fluctuate depending on MSCs’ fate, being higher under osteogenesis conditions, and therefore in osteoblasts [18], and lower in adipogenesis and adipocytes [130]. Subsequently, overexpression of lamin A/C is known to promote osteogenesis differentiation [25], and on the contrary, depletion of lamin A/C levels is associated to a shift to adipogenesis at the expense of osteogenesis by MSCs [30,113,160]. The need of a tight regulation of lamin A/C levels to maintain cellular homeostasis appears evident in the context of aging. Thus, aged mice were shown to have less lamin A/C levels in osteoblasts [161]. This is in agreement with the observation that in aging, the *LMNA* dysregulation is among the mechanisms leading to MSCs’ fate imbalance, increasing adipogenesis at the expense of osteogenesis, a hallmark of age-related bone pathologies [162]. Closely linked to aging, frailty syndrome is usually suffered by older adults, characterized by exhaustion, unintentional weight loss and a multisystemic function decline. Interestingly, frailty patients have been shown to possess low percentages of circulating osteoprogenitors (COPs) [163], cells with characteristics of MSCs [164]. Moreover, a later study proposed lamin A/C as a potential biomarker for this condition, since lamin A/C levels were found to be decreased in COPs from aged individuals suffering frailty [165]. In light of these last evidences, frailty is starting to be considered as a mesenchymal disease and therefore, there are clinical trials addressing MSCs-based therapies to counteract it [166,167].

### 4.2. Premature Aging Syndromes

According to the Universal Mutation Database (www.umd.be/LMNA/), there are over 500 mutations reported on the *LMNA* gene, which lead to at least 15 different laminopathies, a group of rare diseases mainly affecting mesenchymal tissues [168]. Initially, laminopathies were divided into two groups: as tissue-specific disorders, such as striated muscle diseases, lipodystrophic syndromes and peripheral neuropathies, or as multisystem diseases, which affect several tissues, of accelerated aging. However, the observation that there were overlapping clinical phenotypes among different laminopathies [169] suggested that they should be a continuum of related disorders sharing common mechanisms rather than different, independent entities. 

Interestingly, bone pathologies such as skeletal deformities and/or osteoporosis have been described in different *LMNA*-linked accelerating aging syndromes, which differ between them in the time of onset and in the severity of the progeroid phenotypes. Since these premature aging syndromes share an altered lamin A/C protein, it has been proposed that mutated lamin A/C has a negative impact on the homeostasis of MSCs, the natural progenitors of osteoblasts, leading to the observed abnormal bone phenotypes. Next, we will summarize the pathological bone tissue findings in the context of these premature aging laminopathies:

#### 4.2.1. Hutchinson-Gilford Progeria Syndrome (HGPS)

HGPS is a devastating genetic disease, characterized by multiple features of premature aging which mainly affects tissues of mesenchymal origin [170]. Affected children appear healthy at birth, but soon after, exhibit severe growth retardation, lipodystrophy, alopecia and skin changes, among others. Cardiovascular dysfunction usually leads to patients’ death in early teenage years [170]. HGPS is caused by a de novo point mutation in the *LMNA* gene, which activates a splice donor site, resulting in a smaller protein lacking 50 amino acids, among them the cleavage site for the zinc metalloproteinase ZMPSTE24. As a result, the truncated lamin A protein, named as progerin, is permanently farnesylated and therefore tightly associated with the nuclear envelope, leading to nucleus abnormalities [171,172]. Indeed, the nuclear blebbings observed in cells from HGPS patients is considered to be a cellular hallmark of the disease, and to a greater extent, of other laminopathies. HGPS patients develop low bone mass and atypical skeletal geometry, and HGPS is thus considered to be a skeletal dysplasia [173]. Since osteoblasts, the bone forming cells, come from MSCs’ differentiation, an impaired MSCs osteogenesis could be a possible explanation for this bone phenotype in progeria. In fact, MSCs exhibited the higher expression of progerin among different cell types differentiated from progeria-MSCs [174]. Several studies have confirmed this hypothesis: Scaffidi and Misteli were the first addressing this possibility, by the use of human immortalized MSCs stably expressing progerin [175]. Unexpectedly, and taking into account the bone phenotypes of HGPS patients, such as osteoporosis, they showed an increased osteogenesis in MSCs due to progerin accumulation. This result has been later corroborated by other groups using primary human MSCs derived from progeria-iPSCs [176,177]. Interestingly, secretome from MSCs which accumulate prelamin A, another farnesylated precursor of lamin A, whose accumulation is associated with premature aging and bone pathological phenotypes [178], has been shown to be pro-osteogenic, enhancing the osteogenesis of MSCs [179]. The high bone turnover that HGPS patients show has been proposed as an explanation of this “premature osteogenesis” induced by accumulation of unprocessed lamin A precursors in MSCs.

#### 4.2.2. Mandibuloacral Dysplasia Type A (MADA)

There are about 15 different *LMNA* mutations leading to MADA [180], a progeroid laminopathy with mildly accelerating aging and a special affectation of bone and adipose tissue [181]. Regarding bone phenotypes, affected patients usually exhibit osteolysis and osteoporosis, suggesting an increased bone turnover and a defective osteogenesis of MSCs as causative altered processes; however, the studies with primary cells from MADA patients to address this question are scarce. Pre-osteoblasts isolated from one MADA patient carrying a homozygous mutation R527H in the *LMNA* gene showed no impairment in osteogenic differentiation [182]. However, the secretome composition from these MADA-pre-osteoblasts was altered, showing an increased expression of osteoprotegerin and TGF-β2. The MADA-secretome enhanced osteoclast differentiation, which could explain an increased bone turnover and therefore the osteolysis phenotype seen in these patients.

#### 4.2.3. Atypical Progeroid Syndromes (APS)

Over 20 mutations in the *LMNA* gene lead to APS (www.umd.be/LMNA/). Patients affected by atypical progeroid syndromes have variable manifestations of progeroid features, becoming evident in the second or third decade of life. Bone pathologies related to aging have also been described in these patients, such as osteoporosis [183,184,185], and thinning of cortical bone together with femoral fractures in different reported cases of APS [185]. *LMNA* mutations leading to APS do not affect lamin A processing, and therefore, cells isolated from patients have been shown to not accumulate either of the two pathological unprocessed forms of lamin A: progerin or prelamin A [186]. However, these mutations seem to have a role in MSCs’ fate, thus hampering osteogenesis and leading to the described bone phenotypes, but unfortunately, there are not any studies addressing this question. Currently, the underlying mechanisms leading to APS are unknown, but it is likely that the mutation has a negative impact in the dimerization of mutant lamin A/C, and therefore in the proper formation of lamin A/C filament meshwork. This, in turn, would hamper the proper formation of nuclear lamina, disturbing lamin A/C interactions with the other nuclear envelope proteins as well as with transcription factors and chromatin. 

## 5. Conclusions

**Figure 4:**
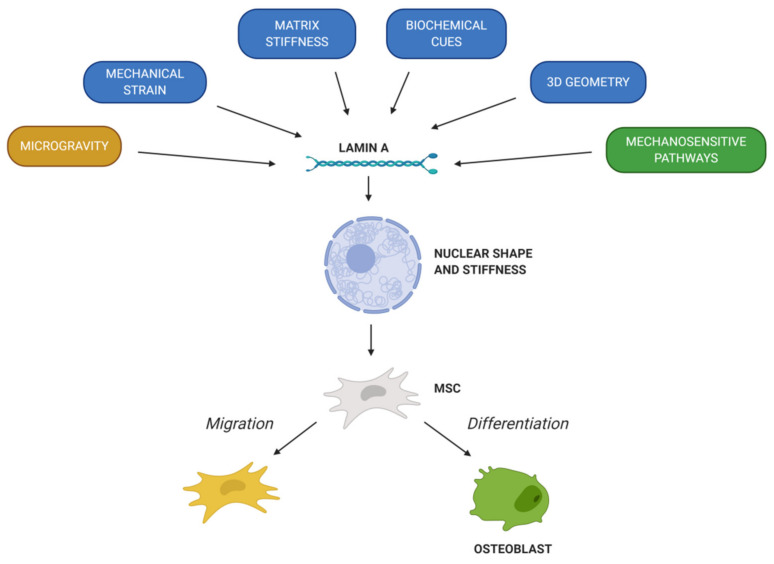
Chemical and mechanical factors that regulate MSCs’ migration and differentiation. These factors regulate lamin A/C levels, thus affecting nuclear shape and stiffness. In this way, MSCs’ migration and/or differentiation into osteogenic lineage depend on lamin A/C expression.

The different characteristics of MSCs pinpoint them as crucial tools to elucidate the mechanisms governing bone repair and disorders. Likewise, as MSCs present many beneficial properties, they are under extensive investigation for their potential in bone therapy. MSCs have demonstrated the capacity to migrate into specific tissues and differentiate into chondrogenic, osteogenic or adipogenic lineage. However, studies in vivo have shown that the migration and differentiation capacity of MSCs is limited, so the expected engraftment is not usually obtained. In this way, factors involved in MSCs’ migration and osteogenic differentiation must be widely studied. Chemical factors (chemokines, cytokines and growth factors) are the ones that induce MSCs’ migration to injured tissue and determinate lineage specification. In addition, mechanical cues play a key role in MSCs’ migration and differentiation. In fact, the nuclear envelope protein lamin A/C regulates the response of MSCs to external factors. First of all, lamin A/C levels determinate the nuclear shape and stiffness of the cell, which is essential to promote MSCs’ migration and cell fate. Then, lamin A/C is linked with the LINC complex, thus connecting cytoskeleton with the nucleus and playing a vital role in mechanotransduction. In summary, mechanical strain, matrix stiffness, 3D geometry and microgravity will regulate MSCs’ migration and differentiation into osteoblasts, thus promoting bone formation and remodeling (Figure 4). Moreover, *LMNA* mutations and level alterations could disrupt MSCs’ fate. Indeed, several *LMNA* mutations affect bone tissue homeostasis and lead to a pathological condition, such as premature aging syndromes. All in all, it is necessary to further delve into the knowledge of lamin A in MSCs’ migration and osteogenic differentiation to understand its implication in bone formation and remodeling; that way, to develop new potential therapies to address bone disorders.

## Abbreviations

Definition:	Abbreviation:
Two-dimensional	2D
Three-dimensional	3D
Atypical progeroid syndromes	APS
Bone marrow mesenchymal stem cells	BMSCs
Bone morphogenetic protein 2	BMP-2
Bone sialo-protein	BSP
Circulating osteoprogenitors	COPs
Cluster of differentiation	CD
Dentin matrix acidic phosphoprotein 1	DMP-1
Extracellular Matrix	ECM
Human leucocyte antigen-DR	HLA-DR
Hutchinson-Gildford progeria syndrome	HGPS
Interleukin	IL
Linker of nucleus and cytoskeletonLow density lipoprotein	LINCLDL
Mandibuloacral dysplasia type A	MADA
Inner nuclear membrane proteinMegakaryoblastic leukaemia 1	MAN-1MKL1
Mesenchymal stem cells	MSCs
Mouse embryonic fibroblasts	MEFs
Nuclear envelope spectrin-repeat-containing proteins	Nesprins
Osteocalcin	OCN
Osteoprotegerin	OPG
Osterix	OSX
Peroxisome proliferator-activated receptor	PPAR
Platelet-derived growth factors	PDGFs
Receptor activator of nuclear factor k-B ligand	RANKL
Retinoic acid	RA
Retinoic acid receptors	RAR
Run-t related transcription factor	RUNX2
Serum response factor	SRF
Sry-related high-mobility group box 9	SOX9
Transcriptional coactivator with PDZ-binding motif	TAZ
Transforming growth factor-β1	TGF-β1
Tumor necrosis factor-α	TNF-α
Vascular endothelial growth factor A	VEGFA
Yes-associated protein	YAP
